# Dynamic Susceptibility Contrast Magnetic Resonance Imaging with Carbon-Encapsulated Iron Nanoparticles Navigated to Integrin Alfa V Beta 3 Receptors in Rat Glioma

**DOI:** 10.3390/nano15161277

**Published:** 2025-08-18

**Authors:** Agnieszka Stawarska, Magdalena Bamburowicz-Klimkowska, Wojciech Szeszkowski, Ireneusz Piotr Grudzinski

**Affiliations:** 1 Department of Toxicology and Food Science, Faculty of Pharmacy, Medical University of Warsaw, Banacha 1, 02-097 Warsaw, Poland; mjbamburowicz@wum.edu.pl; 2 Department of Clinical Radiology, Faculty of Medicine, Medical University of Warsaw, Banacha 1A, 02-097 Warsaw, Poland; wojciech.szeszkowski@wum.edu.pl

**Keywords:** carbon-encapsulated iron nanoparticles, alfa V beta 3 integrin, glioma, magnetic resonance imaging

## Abstract

Overexpression of αvβ3 integrin is found in a diverse group of tumors originating from glial cells in the brain, making this transmembrane receptor a promising biomarker for molecular MRI diagnosis. In the study, we conjugated a monoclonal antibody against the β3 subunit (CD61) of the αvβ3 integrin receptor with carbon-encapsulated iron nanoparticles to yield Fe@C-(CH_2_)_2_-CONH-anti-CD61 bioconjugates that were used in dynamic susceptibility contrast magnetic resonance imaging (DSC-MRI). Wistar rats bearing C6 gliomas were injected as a single bolus (0.5 mL) through the tail vain with a suspension of Fe@C-(CH_2_)_2_-CONH-anti-CD61 nanoparticles (200 μg mL^−1^) and the animals were imaged using the T2*-weighted echo planar imaging (T2* EPI) technique. Results showed that intravenously infused nanoparticles targeting αvβ3 integrin receptors provide strong contrast in rat glioma tissues. No such effects were observed in other rat organs, although some post-contrast effects were also noted in the liver and kidney. The study shows that the as-developed nanoparticles decorated with anti-CD61 monoclonal antibodies might be considered as a novel contrast candidate for noninvasive DSC-MRI diagnosis in CD61-positive gliomas.

## 1. Introduction

Integrins have critical functions in malignancies, angiogenesis, cell invasion and migration and play vital roles in other cancer-relevant processes, including white blood cell trafficking and activation, chronic inflammation and immune mimicry, which ultimately determine disease state [1]. Since integrins are integral to the process of cell adhesion and migration, these transmembrane receptors have been assessed as potential contributors to glioma invasion, as have the co-operating extracellular matrix (ECM) components [1]. The ECM in the perivascular region of the brain comprises such components as collagen, fibronectin and laminin [2]. The perivascular region is a site of frequent invasion, and it has been shown to be a site where tumor cells are capable of adhering and spreading particularly well [3]. Expression of integrin β1 is enhanced in both high-grade glioma samples and in glioma-derived cell lines, relative to normal brain tissue samples [4,5]. The β1 subunit is also a key integrin component as it is able to partner many subunits capable of interaction with the ECM components present in the perivascular region [4]. These integrins typically mediate epithelial cell adhesion to the basement membrane, but might contribute to migration, proliferation and survival in tumor cells [6]. Both αvβ3 and αvβ5 integrins are expressed in astrocytes and epithelial cells at the tumor–normal tissue margin and together with corresponding ECM components have a possible role in invasion, as they can be highly upregulated in cancer tissue [5,7,8,9]. Both the αv and to a lesser extent β3 integrin subunits are expressed in numerous glioma-derived cell lines and high-grade glioma biopsies [6,8,10]. Tumor cell expression of αvβ3, and αvβ5 integrins, is correlated with disease progression in glioblastoma [6,9]. Note that αvβ3 and αvβ5 integrins are associated with the tumor vasculature of anaplastic astrocytomas and glioblastoma multiforme specimens but are not expressed in the vasculature of low-grade astrocytomas or in normal tissue [8]. Furthermore, expression of αvβ3 is observed at the periphery of high-grade gliomas, while, in contrast, expression of αvβ5 was greater towards the center of the tumor [4]. The integrin α6β1 plays an important role in the regulation of glioma-initiating cells [11]. This integrin mediates the interaction of glioma-initiating cells with laminin, an extracellular matrix protein expressed in basement membranes, including those supporting endothelial cells [12]. Such interaction provides an anchorage for glioma-initiating cells within the perivascular niche and supports their tumorigenic potential [5]. As integrins are overexpressed in different glioma cells [13], these molecules make them attractive targets for developing novel nanotechnology-based targeted contrast agents used in magnetic resonance imaging (MRI) [14].

Nanoparticle-targeted imaging has emerged as a promising strategy in diagnostic medicine, particularly in the context of cancer. Functionalized nanomaterials play a pivotal role in advancing biomedical imaging and diagnostics by offering enhanced sensitivity, specificity and multiplexing capabilities. These materials, ranging from noble metal-based nanoparticles (e.g., gold, silver), semiconductor quantum dots and iron oxide nanoparticles, to carbon nanomaterials, are engineered at the nanoscale and functionalized with specific ligands to interact with molecular targets relevant to disease processes [15,16]. The surface modification, or functionalization, of these nanomaterials involves the attachment of biological ligands such as antibodies, aptamers, peptides or small molecules such as folates, and even vitamins or carbohydrates that confer target specificity and biocompatibility [17]. In diagnostic applications, the choice of ligand determines the targeting capacity and functional performance of the nanomaterial. Antibodies, with high affinity and specificity, are frequently used for the detection of protein biomarkers such as HER2 in breast cancer or PSA in prostate cancer [18]. Aptamers—short, single-stranded oligonucleotides—are synthetically selected to bind various targets including small molecules, proteins and cells, and offer advantages such as low immunogenicity and ease of chemical modification [19]. Peptides, due to their small size and stability, are employed for targeting receptors like integrins (e.g., αvβ3) overexpressed in tumor vasculature [20]. Small molecules such as folic acid are also used as targeting ligands, exploiting their affinity for receptors (e.g., folate receptor) that are upregulated in many cancers [17]. All these ligands enable nanomaterials to recognize and bind to a wide range of molecular targets, including cell surface receptors, enzymes, nucleic acids and exosomal markers. Such targeting facilitates the use of nanomaterials in various imaging modalities. For instance, gold nanoparticles conjugated with antibodies or peptides enhance contrast in computed tomography (CT) and photoacoustic imaging due to their strong X-ray attenuation and plasmonic properties [21]. Quantum dots functionalized with aptamers or antibodies serve as bright, photostable probes in fluorescence imaging for the detection of cancer cells or circulating tumor markers [22]. Magnetic nanoparticles such as superparamagnetic iron oxide particles, when functionalized with tumor-targeting ligands, improve the resolution and specificity of magnetic resonance imaging, aiding in early cancer detection and localization [20]. Beyond imaging, functionalized nanomaterials are increasingly integrated into biosensors and point-of-care (POC) diagnostics. Their high surface area and customizable surfaces enable the capture and detection of trace amounts of disease biomarkers in blood, saliva or urine. Nanoparticle-based biosensors employing ligands like aptamers or antibodies can detect molecular targets such as microRNAs, cytokines or viral antigens with high sensitivity and rapid turnaround, making them promising tools for early diagnosis and monitoring [23]. However, despite significant preclinical success, major scientific and translational gaps of using targeted nanoparticles remain that limit their routine clinical use. One of the central challenges is the low delivery efficiency of nanoparticles to target tissues. Quantitative studies have demonstrated that less than 1% of the injected dose of nanoparticles typically accumulates at the target site, with the rest being rapidly cleared by the mononuclear phagocyte system, particularly the liver and spleen [24]. Another limitation lies in the biological behavior of nanoparticles, which is influenced by their interaction with blood components. Upon systemic administration, nanoparticles rapidly adsorb plasma proteins, forming a “protein corona” that can mask targeting ligands and alter biodistribution [25]. This phenomenon often leads to reduced targeting specificity and enhanced clearance, diminishing the effectiveness of active targeting strategies. While many nanoparticle formulations show minimal or moderate toxicity in animal models, the long-term biocompatibility and immunogenic potential in humans are less well understood. Inorganic nanoparticles, such as those based on iron oxide or gold, raise concerns about tissue accumulation and degradation, especially in the absence of reliable clearance mechanisms [26]. From a technical standpoint, imaging sensitivity and quantification present further limitations. Many nanoparticle platforms used in magnetic resonance imaging, for example, require relatively high local concentrations to produce a measurable signal, which increases the required dose and potential toxicity [27]. Finally, the complexity of nanoparticle synthesis, surface functionalization and reproducibility creates substantial barriers to clinical translation. Variability in size, surface charge and ligand density not only impacts biological performance but also complicates regulatory approval. Current good manufacturing practices (GMPs) are not yet fully adapted to the nuanced production requirements of multifunctional nanoparticle systems [28]. While nanoparticle-targeted imaging is a powerful tool with significant theoretical and experimental support, its clinical application is hindered by biological, technical and regulatory limitations. Addressing these challenges will require interdisciplinary approaches that combine advances in nanotechnology, imaging science, pharmacology and regulatory policy to enable the safe and effective use of targeted nanoparticles in medical diagnostics.

Recent advances in nanotechnology have opened new avenues for early diagnosis and treatments of cancers due to functionalized next-generation nanoparticles navigating to different molecular targets [29,30,31]. One of these promising targets are integrins already tested in both preclinical and clinical studies [32,33,34,35]. Types of integrin-targeted nanosized contrasts include superparamagnetic iron oxide nanoparticles [14,36,37], gadolinium-based nanoparticles [38,39,40] and porphyrin-based nanoparticles [37]. Each type has its own advantages and disadvantages. Magnetic nanoparticles (MNPs) have emerged as promising candidates for cancer diagnosis and therapy due to their unique physical properties, such as their small size, magnetic properties and biocompatibility [41,42,43]. Moreover, functionalization of MNPs with ligands, such as monoclonal antibodies, tumor-penetrating peptides and aptamers can enhance their specificity and selectivity towards cancer cells that could be used as targeting contrast agents in MRI [43,44,45].

Magnetic resonance imaging is the most commonly used technique for gliomas due to its superior soft tissue contrast and ability to differentiate between normal and tumor tissues [46,47]. Key features of MRI for glioma diagnosis include T1- and T2-weighted imaging, fluid-attenuated inversion recovery (FLAIR) imaging, diffusion-weighted imaging (DWI), perfusion-weighted imaging (PWI) and magnetic resonance (MRS) spectroscopy [37,39,48,49]. Here, we developed a novel MRI contrast agent composed of carbon-encapsulated iron nanoparticles functionalized with monoclonal antibodies against CD61 (Fe@C-(CH_2_)_2_-CONH-anti-CD61) to target the beta 3 subunit (CD61) of the integrin αvβ3 transmembrane receptor, which is highly overexpressed on glioma cells. Dynamic susceptibility-weighted contrast-enhanced (DSC-MRI) imaging was performed on Wistar rats bearing C6 glioma tumors using monoclonal antibody-navigated nanoparticles as T2*-weighted contrast agents.

## 2. Materials and Methods

Carbon-encapsulated iron nanoparticles were synthesized by a carbon arc discharge route [50,51] and fully characterized for the size, shape, composition and physicochemical properties as described elsewhere in detail [52]. More bioconjugation studies on carbon-encapsulated magnetic nanoparticles using polyclonal and monoclonal antibodies to yield Fe@C-(CH_2_)_2_-CONH-anti-CD61 bioconstructs have been completed and characterized in our previous studies [20,53]. The magnetic and relaxometric properties of carbon-encapsulated iron nanoparticles as a negative contrast agent for MRI have been recently described [54].

In animal studies, anti-CD61 monoclonal antibody-functionalized carbon-encapsulated iron nanoparticles (Fe@C-(CH_2_)_2_-CONH-anti-CD61) were tested as contrast agents using mouse IgG1 anti-rat CD61 monoclonal antibodies (BD Biosciences, San Jose, CA, USA) as navigating ligands. Male Wistar rats (6–7 weeks old, n = 5) purchased from the Mossakowski Medical Research Institute Polish Academy of Science were used in the experiments following the protocol approved by the local ethical committee for experimental animals (No 3/2009). Rat glioma C6 cells (ATCC-CCL-107) suspended in phosphate buffered saline (Sigma-Aldrich, St. Louis, MO, USA) were administered (10^5^ cells per 100 µL^−1^) subcutaneously into the right flank of the animals and the rats bearing C6 tumors were examined three weeks after post-implantation. A 1.5T scanner (Magnetom Avanto, Siemens Medical Solutions, Erlangen, Germany) and a 4-channel surface receiver coil “flex” (Siemens Medical Solutions, Erlangen, Germany) were used in the imaging studies of rats. The imaging protocols were conducted using the T2*-weighted echo planar imaging (T2* EPI) with spoiler in the coronal plane (TR/TE 1250/58 ms, flip angle 90°, FoV 200 mm, slices 8, slice thickness 3 mm, base resolution 128, phase resolution 100%, bandwidth 1502 Hz/Px, EPI factor 128, gradient mode Fast*). Regardless of the adopted EPI regime and acquisition, the T2-weighted (anatomical) turbo spin echo (TSE) sequence in the coronal orientation (TR/TE 3000/80 ms, flip angle 150°, FoV 200 mm, slices 23, slice thickness 2 mm, base resolution 250, phase resolution 100%, bandwidth 191 Hz/Px) was also applied in the studies. All imaging data was analysed at a commercially available workstation (Leonardo workstation for MRI, Siemens, Erlangen, Germany) and BrainMagix version 2.0.1 (Imagilys SPRL, Belgium). The animals bearing C6 glioma under ketamine/xylosin (Sigma-Aldrich, St. Louis, MO, USA) anesthesia (45/3 mg kg^−1^) were placed in the MR scanner (1.5T). The MRI examination began with setting the so-called localizer sequence and precise positioning of the animal in the 3D planes. In order to precisely dose the nanoparticle suspension to the tail vein of rats, automatic administration was applied from a dispenser programmed in a defined time cycle. In the studies, the nanoparticle suspension was prepared in a physiological sterile phosphate buffered saline (PBS) solution, to which carboxymethylcellulose (CMC, Sigma-Aldrich, St. Louis, MO, USA), as a surfactant, was added at a concentration of 0.1 mg mL^−1^. The as-prepared suspension of Fe@C-(CH_2_)_2_CONH-anti-CD61 nanoparticles (200 μg mL^−1^) was administered in a single rapid bolus (0.5 mL) through a cannulated catheter into the tail vein of rats bearing C6 glioma with acquisitions of T2*-weighted EPI images performed in the first 12.5 min (750 s) post-dosing. The anatomical T2-weighted imaging was also performed using TSE sequences. The superimposition of T2*- and T2-weighted images was performed by reconstructing perpendicular sections from the volume in a common coordinate system using image data interpolation. The mutual position and geometry of the volumes as well as the coefficients correcting the shifts and rotation between the collected image series were taken into account. Regions of interest (ROI) were marked on the obtained images, from which an averaged curve of relative arbitrary units of nanoparticle concentration over time was calculated, specifying the time to reach the maximum amplitude on the pharmacokinetic curves. The MR signal intensity was converted to a relative concentration of nanoparticles expressed as arbitrary units according to the formula [55]:

(1) $$C_{t}\left(t\right)=k_{t}\Delta R_{2}^{*}\left(t\right)=-\frac{k_{t}}{TE}ln\left(\frac{S(t)}{S_{0}}\right)$$

where:

*C*_t_(*t*)—the relative concentration of the contrast agent (nanoparticles) in the tissue at time *t*;

*K*_t_—a proportionality constant that depends on the tissue, the contrast agent (nanoparticles), the field strength and the pulse sequence parameters;

*R*_2_*—the relaxation rate (*R*_2_* = 1/T_2_*);

*TE*—the echo time of the MRI sequence;

*S*(*t*)—the signal intensity in the tissue at time *t*;

*S*_0_—the signal intensity during the baseline period before the arrival of the contrast agent (nanoparticles).

## 3. Results and Discussion

Dynamic susceptibility contrast magnetic resonance imaging (DSC-MRI), commonly referred to as the “bolus tracking” method, monitors the first pass of an intravascular, non-diffusible contrast agent through tissue. It relies on susceptibility-induced signal loss observed in T2*-weighted sequences, which results from a bolus of a contrast passing through the capillary bed [56]. This method involves intravenous injection of a paramagnetic or superparamagnetic contrast agent in a bolus, i.e., a single rapid administration, and measurement of the signal during its direct flow [57]. Since the flow time of the contrast agent through the given tissue usually lasts several seconds, the use of fast MR imaging techniques is required for correct characterization of the signal intensity. Echo planar imaging (EPI) and fast low tilt angle sequences are commonly used in such studies [58]. The T2*-weighted sequences based on perfusion measurement are used in the case of contrast agent movement only in the lumen of the blood vessel [59]. For this reason, they commonly use blood pool contrast agents to modulate the signals [60]. Although the vascular space is a small part of the total tissue volume (e.g., for the human brain it is ca. 3–5%), the effect of contrast susceptibility of the flowing agent in the bolus causes a strong momentary decrease in the signal in this space [37]. This type of phenomenon was considered appropriate to analyze the possibility of using innovative core-shell type carbon-encapsulated iron nanoparticles, which in light of the conducted previous studies was classified as a typical negative contrast agent, i.e., reducing the MRI signal intensity on T2- and T2*-weighted magnetic resonance images [52].

**Figure 1:**
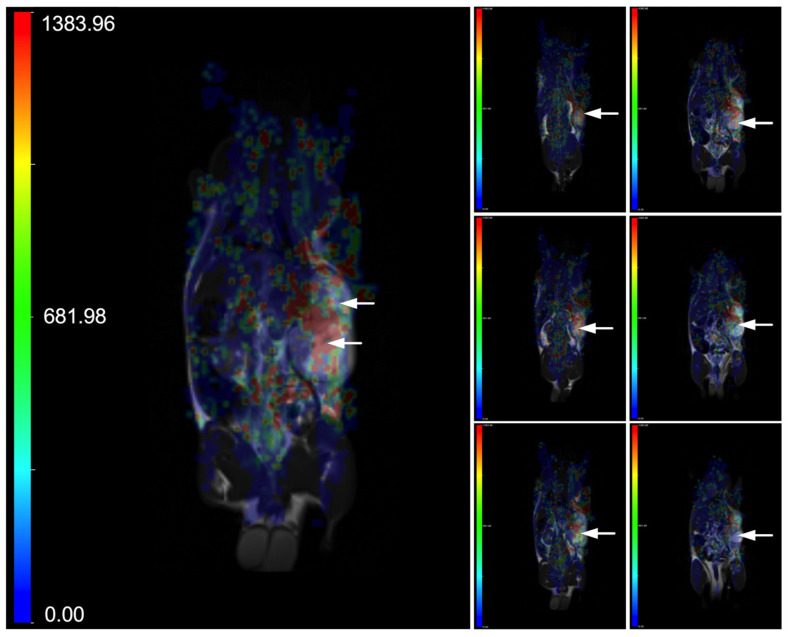
Representative parametric maps of blood volume, overlaid on T2-weighted images, are shown across different slices of a Wistar rat bearing a C6 glioma. The animal underwent dynamic susceptibility contrast MR imaging (DSC-MRI) following intravenous injection of Fe@C-(CH_2_)_2_CONH-anti-CD61 nanoparticles (200 μg mL^−1^) administered as a single bolus (0.5 mL) into the tail vein. DSC-MRI was conducted over 12.5 min using T2*-weighted EPI sequences (TR/TE = 1250/58 ms). Anatomical T2 images were performed using TSE sequences (TR/TE = 3000/80 ms). The left large image displays a parametric map from a single slice, while the right small images combine maps from multiple slices of the same rat. The accumulation of nanoparticles targeting the beta 3 subunit (CD61) on the integrin αvβ3 receptors in glioma tissue leads to localized magnetic susceptibility changes, visualized as areas of increased red intensity on the arbitrary unit (a.u.) color scale (refer to the scale for interpretation). Both the left and right images use the same color scale ranged from 0 to 1383.96 a.u. The C6 glioma is indicated by arrows.

The DSC-MRI study was performed on Wistar rats bearing C6 tumors growing on a flank that were intravenously administered into the tail vain with a single bolus of Fe@C-(CH_2_)_2_-CONH-anti-CD61 nanoparticles conjugated with monoclonal antibodies against the beta 3 subunit (CD61) of the integrin αvβ3 receptors. Note that the sedimentation conditions of the as-used nanoparticle suspension were discussed in the publication by Grudzinski et al. [61]. Based on the performed studies, the targeted deposition of nanoparticles with a functionalized antibody against the CD61 integrin in tumor tissues was demonstrated. This was noted as a strong post-contrast signal change in magnetic susceptibility visible as increased red color on T2*-weighted images (Figure 1). Results shown in Figure 1 provide T2* EPI-weighted images expressed as blood volume parametric maps superimposed with T2-weighted images to better localize the tumor tissues. Creating a blood volume parametric map (Figure 1) from DSC-MRI involved a structured series of image-processing and signal-modeling steps. The process begins with the acquisition of DSC-MRI data during the injection of a nanoparticle-based contrast agent. Once the data were acquired, preprocessing was performed to ensure the accuracy of the signal analysis. This included motion correction to align all volumes over time, and registration to anatomical images. Following preprocessing, the MRI signal was converted into a concentration-time curve by calculating the change in transverse relaxation rate (please see the formula). The relative blood volume was then calculated by integrating the Δ*R*_2_*(*t*) curve over time. This step was performed using numerical methods. Finally, the calculated blood volume values were used to generate a parametric map that matches the spatial dimensions of the original MRI data. The result is a voxel-wise representation of blood volume, useful for assessing C6 glioma perfusion in Wistar rats (Figure 1).

**Figure 2:**
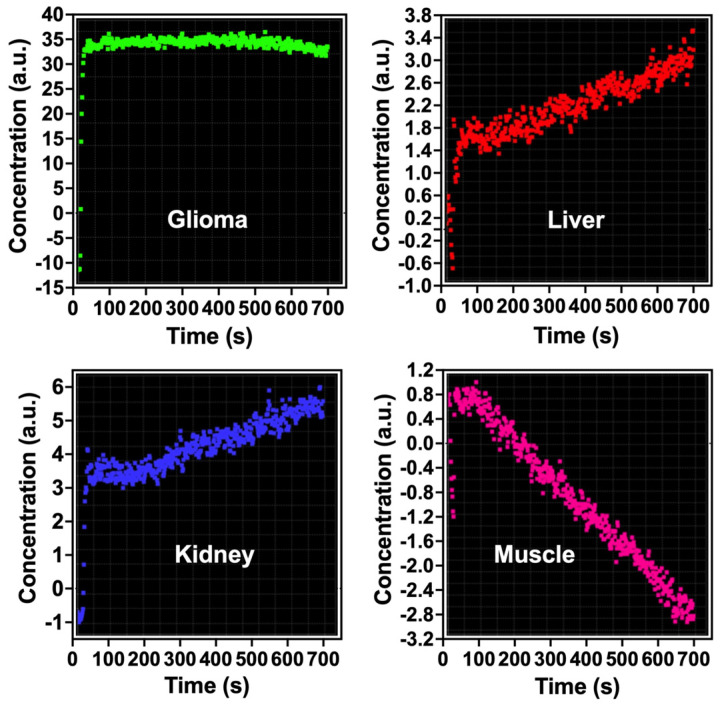
Pharmacokinetic analysis of antibody-conjugated carbon-encapsulated iron nanoparticles (Fe@C-(CH_2_)_2_CONH-anti-CD61) in rat C6 glioma and other organs. The concentration of nanoparticles is reported in arbitrary units (a.u.). A single bolus dose (0.5 mL) containing 200 μg mL^−1^ of the nanoparticles was administered via a cannulated tail vein catheter to rats implanted with C6 glioma. MRI scans were conducted for up to 12.5 min (750 s) post-injection. Dynamic susceptibility contrast MRI (DSC-MRI) was performed using a T2*-weighted EPI sequence with parameters TR/TE = 1250/58 ms.

In the studies, we observed a pronounced increase in the concentration of Fe@C-(CH_2_)_2_CONH-anti-CD61 nanoparticles in glioma tissues and this could be associated with enhanced permeability and retention (EPR) effects (Figure 2). Such a significant increase (arbitrary units) was not found in the case of the liver or kidney (Figure 2). Please note that vascular extravasation in tumors refers to the leakage of blood components, including nanoparticles, from the tumor vasculature into the surrounding tumor tissue due to the abnormal structure and permeability of tumor blood vessels [62]. This phenomenon is often enhanced in tumors, allowing for greater penetration of nanoparticles, which can accumulate at higher concentrations in the tumor microenvironment, making them potential vehicles for targeted drug delivery [63]. The EPR effect, a hallmark of tumors, further facilitates the preferential accumulation of nanoparticles in tumor tissues, improving diagnostic and therapeutic outcomes [64,65]. This process is influenced by factors like nanoparticle size, tumor characteristics and the presence of leaky vasculature. The presence of beta 3 integrin receptors in glioma tissues and glioma vasculature may significantly enhance the deposition of these nanoparticles in glioma as compared to other analyzed organs (Figure 2). Our previous relaxometric measurements indicate the temporary deposition of Fe@C-(CH_2_)_2_CONH-anti-CD61 in glioma and melanoma tumors examined in murine syngeneic mice models [20,54]. The current results therefore confirm the observed effects, indicating the deposition of nanoparticles functionalized with antibodies recognizing CD61 integrin receptors. Of course, in the case of nanoparticles, we cannot rule out the nanoparticle extravasation effects.

**Figure 3:**
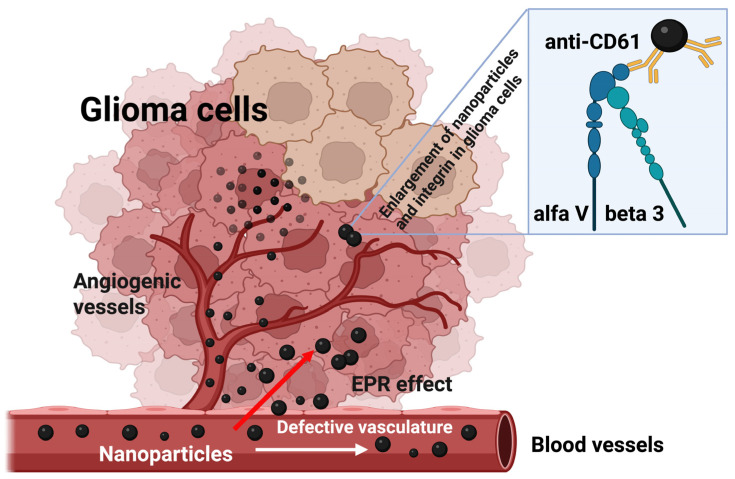
Schematic diagram of nanoparticle extravasation in rat C6 glioma tissues. A single bolus of carbon-encapsulated iron nanoparticles conjugated with a monoclonal antibody (anti-CD61) was injected into the tail vein of Wistar rats bearing C6 glioma. These nanoparticles exited the glioma vasculature through an EPR (enhanced permeability and retention)-mediated effect. Upon reaching the glioma cells, they bound to the αvβ3 integrin, resulting in a detectable change in the magnetic resonance signal (refer to Figure 1). As a result, the concentration of nanoparticles within the glioma tissue increased (refer to Figure 2).

Quantification of bolus-tracking data using an intravascular agent is based on the principles of tracer kinetics for non-diffusible tracers [55]. These are used to model the time-dependent concentration of the nanoparticle-based contrast agent in the tissue as a function of the injected bolus, the blood flow and the fraction of the contrast agent remaining in the tissue. One of the differences of MRI compared with other imaging modalities is that MRI does not measure the concentration of the contrast agent directly. In order to apply pharmacokinetic models of contrast distribution to imaging-based data, the first essential step is to use the signal changes observed in the dynamic acquisition to calculate quantitative parametric images of contrast concentration at each time point. The concentration is related to its effect on the relaxation time and the change (compared with the pre-injection baseline value) in the relaxation rate (*R*_2_* = 1/T_2_*) is linearly proportional to the concentration of the contrast agent (please see the formula) [55]. Pharmacokinetic studies evidenced a rapid increase in the concentration of Fe@C-(CH_2_)_2_CONH-anti-CD61 nanoparticles in the tumor environment at the first minutes following saturation of this process in the next minutes (Figure 2). Minimal post-contrast effects were also observed in the liver and kidney, but the contrast effects were significantly weaker compared to glioma tissues (Figure 2). Basically, no such changes in the resonance signal were observed in the gastrocnemius muscle of the rat, which served as a control for the pharmacokinetic study. When analyzing the concentration profile of antibody-conjugated carbon-encapsulated iron nanoparticles over time in rat C6 glioma tissues (Figure 2), it should be emphasized that no typical curve pattern was observed, such as the one characteristic of gadolinium-based chelate agents routinely used in clinical DSC-MRI studies with glioma patients [66,67]. When gadolinium passes through the blood vessels, it causes changes in the magnetic properties between the vessels and the nearby tissue. Even though blood vessels make up only a small percentage of the total glioma tissue volume, the presence of gadolinium inside them disrupts the uniformity of the local magnetic field. This disturbance spreads beyond just the blood vessels, affecting nearby areas of tissue. As a result, both the spins inside the blood vessels and those in the surrounding tissue experience a drop in T2*, leading to a noticeable temporary decrease in signal. Following a standard intravenous bolus injection, gadolinium typically exhibits a concentration-time curve marked by an initial rise in concentration, followed by a rapid decline as the agent exits the tumor vasculature [68]. In contrast, the nanoparticle concentration profile in our study showed a markedly different pattern (Figure 2). In the glioma tissue, we observed a sharp initial increase in concentration, which then stabilized, forming a sustained plateau lasting up to 750 s. This result clearly shows that nanoparticles are capable of causing blood vessels to leak, a process linked to the phenomenon of extravasation (Figure 3). Additionally, their ability to bind to integrin αvβ3 receptors due to navigating antibodies (anti-CD61) on both the endothelial cells of glioma blood vessels and glioma cells enhances their accumulation within the tumor site. This accumulation may lead to localized decreases in signal intensity on T2*-weighted MRI scans (Figure 1). Notably, this behavior was not observed in the liver or kidneys. Please note that the liver acts as a major site for the capture, processing and elimination of particles, including nanomaterials, which can be taken up by hepatocytes, Kupffer cells and endothelial cells in the liver sinusoids. The fenestrated endothelial architecture of hepatic sinusoids further facilitates nanoparticle entry into the liver parenchyma. This unique permeability allows nanoparticles—particularly those under 100 nm—to diffuse into the space of Disse and be taken up by hepatocytes and stellate cells. Over time, this can lead to nanoparticle accumulation and potential hepatic toxicity, especially for inorganic nanomaterials that are not easily degraded or excreted [69]. Kupffer cells comprise the largest population of tissue macrophages in the organism and play a crucial role in the immune response. Iron nanoparticles are taken up by Kupffer cells and this is used in imaging the primary liver tumor [70]. Interestingly, uptake of iron oxide nanoparticles by Kupffer cells is used as a biomarker in MRI distinguishing benign from malignant liver lesions [71,72]. Renal clearance is another significant pathway for nanoparticle elimination, especially for particles with hydrodynamic diameters below ~5–8 nm. These ultrasmall nanoparticles can pass through the glomerular filtration barrier and be excreted in the urine. However, this is not always beneficial. In many cases, renal clearance competes with the accumulation of nanoparticles at disease sites, shortening circulation time and reducing imaging efficacy. Moreover, repeated exposure or improper surface functionalization can result in tubular reabsorption or nephrotoxicity, particularly if the particles contain heavy metals or reactive surface chemistries [73]. Importantly, the balance between hepatic and renal clearance is strongly influenced by the physicochemical properties of nanoparticles, including size, shape, surface charge and coating composition. For example, larger particles (>100 nm) tend to be retained in the liver and spleen, while smaller, neutrally charged and hydrophilic particles are more likely to be filtered by the kidneys. These competing clearance mechanisms create a narrow design window for optimizing targeted imaging agents that can circulate long enough to reach target tissues, yet avoid premature clearance and off-target accumulation [74]. The challenge, therefore, is not simply reducing liver and kidney uptake, but engineering nanoparticles that can evade these organs long enough to engage disease targets while maintaining biocompatibility and eventual excretion.

Monoclonal antibody-conjugated carbon-encapsulated iron nanoparticles targeting the beta 3 subunit (CD61) of the integrin αvβ3 receptors represent a promising class of nanomaterials for targeted MRI diagnostics. In the context of MRI, these nanoparticles act as effective T2 contrast agents due to their high magnetic susceptibility, significantly improving image contrast and sensitivity [52]. Their superparamagnetic behavior at room temperature minimizes remanence, reducing the risk of particle aggregation—a key factor in safe in vivo applications [52]. Additionally, the carbon shell offers a versatile platform for functionalization with targeting ligands (e.g., CD61), enabling specific binding to biomarkers or tumor cells, thereby enhancing diagnostic precision [20,54]. In bolus-tracking methods such as DSC-MRI, the T2*-based techniques for measuring perfusion using contrast agents are primarily used in cases where there is a significant compartmentalization of the contrast agent (to observe a significant decrease in T2* relaxation). In the present studies, we evidenced nanoparticle extravasation effects, which refers to the movement of nanoparticles from blood vessels into surrounding glioma tissues (Figure 3). This effect is primarily influenced by the EPR effect, where leaky tumor vasculature and poor lymphatic drainage allow nanoparticles to accumulate in tumor tissues more than in normal tissues. While this mechanism enhances nanoparticle accumulation, it may also cause off-target effects and plausibly toxicity in non-tumor tissues. Therefore, understanding and optimizing extravasation is crucial for improving the efficacy and safety of nanoparticle-based diagnostics, and ongoing research will seek nanoparticles for controlled and targeted extravasation.

## 4. Conclusions

The performed preclinical DSC-MRI studies indicate local T2*-weighted contrast changes in magnetic susceptibility in rat C6 glioma tissues after administration of carbon-encapsulated iron nanoparticles functionalized with a monoclonal antibody (anti-CD61) against the beta 3 subunit of the rat integrin αvβ3 receptors. The pharmacokinetic studies of nanoparticles in rats bearing C6 tumors indicate the possibility of nanoparticle accumulation in the glioma tissue. Considering the intravenous administration of nanoparticles in rats, it cannot be ruled out that hepatic deposition also occurred after administration. The studies indicate the importance of surface functionalization of carbon-encapsulated iron nanomaterials with monoclonal antibodies selectively recognizing the beta 3 subunit of the αvβ3 integrin receptor in targeted dynamic susceptibility contrast magnetic resonance imaging in CD61-positive glioma cells. Therefore, the obtained results open new avenues in the field of the use of targeted core-shell type magnetic nanoparticles in both glioma and plausibly liver tumors, because the uptake of these nanoparticles in the hepatic macrophage system may probably be a beneficial element in the case of hepatotropic imaging, including primary liver tumors with overexpression of the integrin receptor αvβ3 [70,75]. Overall, monoclonal antibody (anti-CD61)-conjugated carbon-encapsulated iron nanoparticles hold significant promise in advancing MRI-based diagnostics, offering a combination of high contrast capability and targetability. Further research will determine their preclinical safety and viability in clinical settings. Challenges will also remain in large-scale synthesis with controlled size and surface properties, as well as in thorough long-term stability, extravasation and clearance studies.

## Data Availability

The original contributions presented in this study are included in the article. Further inquiries can be directed to the corresponding author(s).

## Abbreviations

The following abbreviations are used in this manuscript:

MRI	magnetic resonance imaging
DSC-MRI	dynamic susceptibility contrast magnetic resonance imaging
T2* EPI	T2*-weighted echo planar imaging
DWI	diffusion-weighted imaging
PWI	perfusion-weighted imaging
TSE	turbo spin echo
ROI	regions of interest
CD61	beta 3 subunit of the αvβ3 integrin receptor
ECM	extracellular matrix
MNPs	magnetic nanoparticles
TPP	tumor penetrating peptides
EPR	enhanced permeability and retention effect
CT	computed tomography
POC	point-of-care
GMP	good manufacturing practice
